# Extracellular Vesicles as Mediators of Endothelial Dysfunction in Cardiovascular Diseases

**DOI:** 10.3390/ijms26031008

**Published:** 2025-01-24

**Authors:** Francisco Rafael Jimenez-Trinidad, Sergi Calvo-Gomez, Manel Sabaté, Salvatore Brugaletta, Victoria Campuzano, Gustavo Egea, Ana Paula Dantas

**Affiliations:** 1 Department of Biomedical Sciences, School of Medicine and Health Sciences, University of Barcelona, 08036 Barcelona, Spain; frajimenez@recerca.clinic.cat (F.R.J.-T.); vcampuzanou@ub.edu (V.C.); gegea@ub.edu (G.E.); 2 Institut Clínic Cardiovascular (ICCV), Hospital Clínic, 08036 Barcelona, Spain; masabate@clinic.cat (M.S.); sabrugal@clinic.cat (S.B.); 3 Division of Respiratory, Cardiovascular and Renal Pathobiology and Bioengineering, Institut d’Investigacions Biomèdiques August Pi i Sunyer (IDIBAPS), 08036 Barcelona, Spain; 4 Department of Biomedical Sciences, School of Medicine, Universitat Internacional de Catalunya (UIC), 08195 Barcelona, Spain; sergicalvo@uic.es; 5 Rare Diseases Biomedical Research Network Center (CIBERER), Instituto de Salud Carlos III, 28222 Madrid, Spain; 6 Center of Medical Genetics, University of Antwerpen, 2659 Edegem, Belgium

**Keywords:** extracellular vesicles, exosomes, intercellular communication, endothelium, cardiovascular disease

## Abstract

This comprehensive review aims to provide a thorough overview of the vital role that extracellular vesicles (EVs) play in endothelial dysfunction, particularly emphasizing how physiological factors—such as sex and aging—along with significant cardiovascular risk factors, influence this process. The review covers studies ranging from the first description of EVs in 1945 to contemporary insights into their biological roles in intercellular signaling and endothelial dysfunction. A comprehensive analysis of peer-reviewed articles and reviews indexed in the PubMed database was conducted to compile the information. Initially, Medical Subject Headings (MeSH) terms included keywords aimed at providing general knowledge about the role of EVs in the regulation of endothelial signaling, such as “extracellular vesicles”, “endothelium”, and “intercellular signaling”. Subsequently, terms related to the pathophysiological implications of EV interactions with endothelial dysfunction and cardiovascular disease were added, including “cardiovascular disease”, “sex”, “aging”, “atherosclerosis”, “obesity”, and “diabetes”. Additionally, the potential applications of EVs in cardiovascular disease were explored using the MeSH terms “extracellular vesicles”, “cardiovascular disease”, “biomarker”, and “therapeutic strategy”. The results of this bibliographical review reveal that EVs have the capacity to induce various cellular responses within the cardiovascular system and play a significant role in the complex landscape of endothelial dysfunction and cardiovascular disease. The composition of the EV cargo is subject to modification by pathophysiological conditions such as sex, aging, and cardiovascular risk factors, which result in a complex regulatory influence on endothelial function and neighboring cells when released from a dysfunctional endothelium. Moreover, the data suggest that this field still requires further exploration, as EV biology is continuously evolving, presenting a dynamic and engaging area for research. A deeper understanding of the molecular cargo involved in EV–endothelium interactions could yield valuable biomarkers for monitoring cardiovascular disease progression and facilitate the development of innovative bioengineered therapeutic strategies to enhance patient outcomes.

## 1. Introduction

The endothelium, the monolayer of endothelial cells (ECs) lining the inner surface of blood vessels and capillaries, is a marvel of complexity. It transcends its traditional role as a passive barrier between the bloodstream and tissues, functioning as a highly active metabolic, endocrine organ, releasing a diverse array of vasoactive substances, inflammatory mediators, growth factors, and regulators of coagulation [1,2,3]. Groundbreaking studies by Furchgott and Zawadzki on the aorta (1980), followed by studies by Carvalho and Furchgott on arterioles (1981), were the first to unveil the critical role of the endothelium in modulating vascular tone [4,5]. Since then, extensive research has established that the endothelium is not just a barrier but a dynamic organ essential for maintaining circulatory homeostasis, regulating vascular tone, coagulation cascade, and angiogenesis.

One of the most intriguing aspects of the endothelium is the plasticity of its cells. ECs can adapt their phenotype in response to local environmental cues. Under physiological conditions, this adaptability supports tissue repair and maintains blood flow, thereby contributing to hemostasis. However, under pathological conditions, EC maladaptation can lead to a dysfunctional endothelium, a state characterized by functional abnormalities and significant structural alterations, such as nuclear vacuolization, cytoplasmic edema, cell fragmentation, and loss of adhesion to the extracellular matrix [6,7].

Endothelial dysfunction is a pivotal contributor to the onset and progression of various diseases. It is defined by a dysregulated release of endothelium-derived factors, leading to reduced vasodilatory capacity, intensified pro-inflammatory and pro-thrombotic states, and the abnormal modulation of vascular growth. In cardiovascular diseases (CVD), endothelial dysfunction is a central player in the pathophysiology of atherosclerosis, promoting vascular stiffness, reducing arterial distensibility, and exacerbating damage to both the vasculature and myocardium [8,9]. Despite their clinical importance, the precise molecular mechanisms that trigger and sustain endothelial dysfunction remain incompletely understood. Known risk factors, including an imbalance in oxidative stress, increased blood pressure, hyperglycemia, dyslipidemia, exposure to environmental pollutants, chemical agents, and aging, can contribute to the onset or progression of endothelial dysfunction [10,11,12,13]. The early stages of endothelial dysfunction involve subtle, subclinical changes in EC behavior; however, its progression triggers a cascade of events that lead to vascular remodeling and cardiovascular deterioration, ultimately contributing to the development of CVD [13].

**Figure 1:**
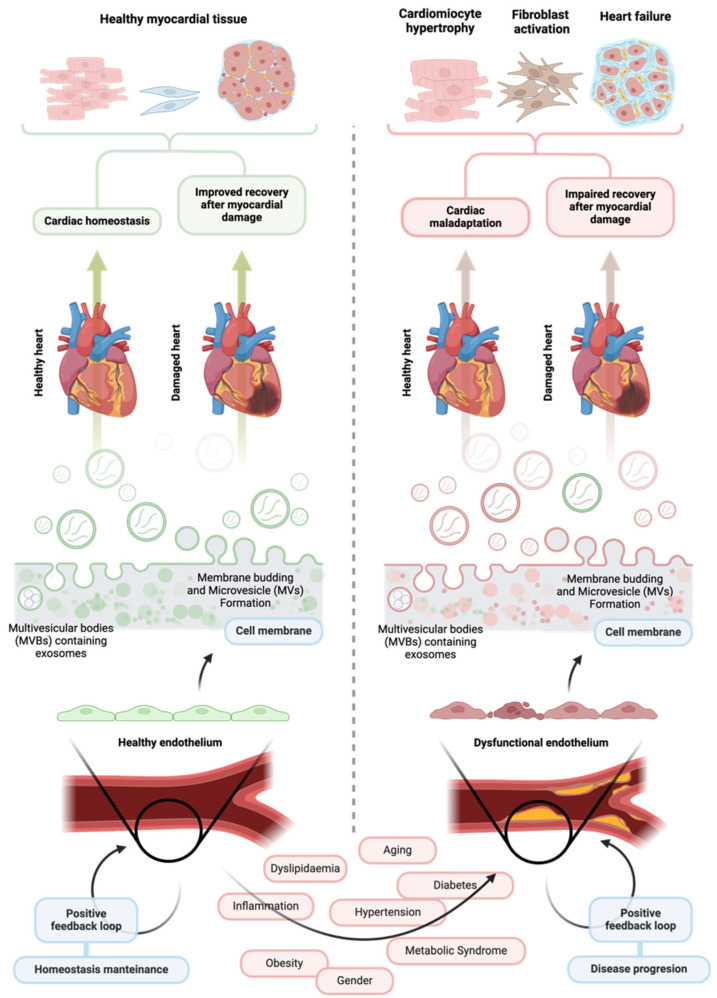
The influence of healthy and dysfunctional vascular endothelium and their extracellular vesicles on cardiovascular health. A healthy vascular endothelium is crucial for maintaining cardiac stability and overall cardiovascular health. Endothelial cells release various signaling molecules to the intercellular space, including extracellular vesicles carrying molecular cargo capable of positively influencing the function of surrounding cells in the cardiovascular system. Through this process, the endothelium can help maintain cardiac homeostasis and facilitate effective repair after myocardial infarction. In contrast, extracellular vesicles released by a dysfunctional endothelium may contain a different molecular cargo that can worsen endothelial dysfunction and negatively impact the function of neighboring cardiovascular cells. These effects can severely and adversely affect heart morphology, ultimately hindering recovery following ischemic injury and undermining cardiovascular health. Created at https://BioRender.com.

ECs communicate extensively with surrounding cells through a rich and complex secretome. This interaction involves various forms of signaling, from localized ion exchange to the release of biochemical messengers, facilitating environmental adaptation and functional regulation [14,15]. Recently, a novel form of intercellular communication has been identified for endothelium-mediated intercellular communication: extracellular vesicles (EVs). These membrane-bound micro and nanostructures have emerged as key players in cell signaling, acting as carriers of biological information that can modulate the phenotype and function of recipient cells. In this review, we will explore the role of EVs as critical regulators of endothelial function, highlighting their potential contribution to endothelial dysfunction and associated cardiovascular complications [16] (Figure 1).

## 2. Extracellular Vesicles: Biogenesis, Characteristics and Signaling Mechanisms with Target Cells

EVs are a heterogeneous group of small lipid-bound particles that hold the potential to revolutionize our understanding of intercellular communication. Secreted by cells into the extracellular environment, they carry a cargo of bioactive molecules such as proteins and non-coding RNAs (ncRNAs), including micro-RNA (miRNA), long non-coding RNA (lncRNA), and circular RNA (circRNAs) from the donor cell, which can be transferred between cells to mediate intercellular communication [17].

The discovery of EVs dates back to 1945 when Chargaff’s experiments on blood coagulation identified a high-speed sediment that altered clotting times [18]. In a follow-up study, Chargaff’s group hypothesized that these sediments might be fragments of blood cells, although they also speculated that they may have formed during the isolation process [19]. From the 1960s to the 1980s, with the advent of electron microscopy, multiple independent researchers observed vesicular structures in exocytotic bodies released by cells into the extracellular space [20]. However, it was not until the early eighties that two independent laboratories published groundbreaking papers linking transferrin receptors on reticulocytes to small vesicles, which were then termed exosomes [21,22].

Since their foundational discoveries, extensive research has been carried out to expand our understanding of EV biogeny, characteristics, and function. Studies have described their biogenesis mechanism, size distribution, surface markers, molecular content, and functions in both physiological and pathological contexts. Moreover, advances in methodologies have also significantly improved the protocols for isolation and characterization, although challenges remain [17].

EVs are categorized into two main types of particles based on their biogenesis, size, and surface markers. Although other vesicle-like entities, such as apoptotic bodies and oncosomes, share some similarities with EVs, they are not traditionally classified as such. For instance, apoptotic bodies are larger and released during programmed cell death, while oncosomes are larger and released by cancer cells. These distinctions are important to note as they help to avoid confusion and ensure a clear understanding of EVs. For this reason, these particles (apoptotic bodies and oncosomes) will not be covered in this review.

**Figure 2:**
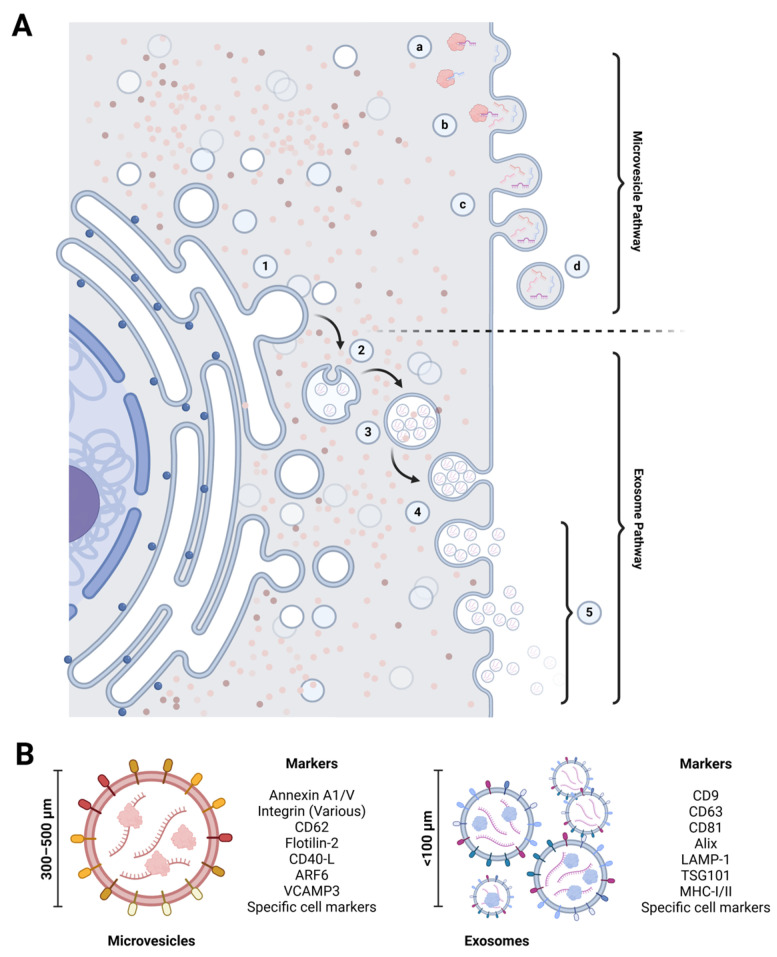
Mechanisms of extracellular vesicle biogenesis, exocytosis, and defining characteristics. (**A**) Extracellular vesicle biogenesis: the formation of macrovesicles begins with the accumulation of bioactive compounds beneath the cell membrane (a, b), which make up the vesicle cargo. This process is followed by membrane budding and constriction (c), resulting in the release of evaginated microvesicles into the extracellular space (d). In contrast, exosome biogenesis occurs within the endoplasmic reticulum. It involves the formation of intraluminal vesicles through membrane invagination (1, 2, 3). These intraluminal vesicles become exosomes when the multivesicular body fuses with the cell membrane (4) and release them into the extracellular space (5). (**B**) Extracellular vesicle biomarkers: microvesicles and exosomes have specific membrane markers that aid their identification and characterization. These markers differ based on the type of extracellular vesicle and their cellular origin. Created at https://BioRender.com.

The larger entities, named microvesicles (MVs), are formed through the outward budding of the plasma membrane, typically resulting in EVs ranging from 0.1 to 1 µm in diameter. They display distinct membrane markers such as CD40 and various integrins and selectins, contributing to their functional roles. Exosomes, on the other hand, are nanosized vesicles (approximately 40–120 nm) originating from the invagination of endosomal membranes, leading to the formation of multivesicular bodies (MVBs) characterized by specific surface markers like CD9, CD63, and CD81 [23]. Upon fusion of MVBs with the cell membrane, exosomes are released into the extracellular space (Figure 2). Specific characteristics are often used to differentiate between MVs and exosomes; however, it is important to recognize that no definitive cutoff clearly defines these entities. A study’s design and methodology, implemented for purification and characterization, may influence the variability in their features and blur the distinctions between larger exosomes and smaller MVs. This overlap indicates that not all EVs will consistently contain the same markers and concentrations. Nevertheless, both types of EVs are essential in facilitating intercellular communication in various physiological and pathological contexts, even though their cargo—especially miRNAs—and expected roles can differ significantly. Understanding these nuances is crucial for advancing research and therapeutic applications involving EVs.

After being released from donor cells, EVs engage in complex and dynamic interactions with recipient cells through various pathways. The lipid composition of both EVs and the membranes of recipient cells facilitates multiple mechanisms of EV entry, including endocytosis and phagocytosis. Alternatively, EVs may fuse directly with lipid rafts in the plasma membrane of recipient cells, releasing their bioactive contents into the cytoplasm. Once inside the cell, the molecular cargo of EVs can lead to significant changes in the activities of intercellular proteins and gene expression patterns, ultimately altering the functional state of the recipient cells [24,25].

In addition to their internalization capabilities, EVs carry surface proteins that can specifically interact with receptors on recipient cells. This interaction is crucial as it can trigger cascades of intracellular signaling pathways that profoundly influence cellular behavior. This interaction is pivotal, as it can initiate cascades of intracellular signaling pathways that significantly influence cellular behavior. For instance, direct engagement of EVs with membrane-bound morphogens, such as Wnt proteins and the Notch ligand DII4, can activate various downstream signaling pathways that regulate essential biological processes, including cell proliferation, migration, and differentiation [24,25]. Furthermore, EVs can play a crucial role in the modulation of immune responses, thus contributing to the regulation of inflammation—a vital aspect of cellular communication. By carrying immunomodulatory molecules, such as cytokines and other signaling factors, EVs can influence the behavior of immune cells. Depending on the pathophysiological context, EVs may promote or suppress immune responses, thereby shaping the overall immune landscape and affecting tissue homeostasis [24,25].

## 3. Extracellular Vesicles and Intercellular Communication in the Endothelium

Emerging evidence strongly indicates that EVs play a pivotal role in modulating ECs, influencing both their regulation and dysfunction. Not only are ECs targets for circulating EV-mediated regulation, but they also serve as vital contributors to intercellular communication in different organs by releasing their own EVs to neighboring tissues.

The endothelium is recognized as the second largest source of circulating MVs in the bloodstream, following the dominant contribution from platelets. Endothelial-derived EVs are increasingly recognized as reliable biomarkers of endothelial health and injury and key players in the complex mechanisms underlying vascular diseases. These vesicles are carriers of bioactive molecules, including proteins, lipids, and RNAs, which affect recipient cells depending on the surrounding context. The evident diversity within EVs’ molecular cargo is influenced by a range of factors, including the process of their biogenesis, the specific type of cell from which they originate, and the physiological or pathological state that the originating cell is undergoing. It is proposed that this finely tuned cargo-loading mechanism enables cells to selectively eliminate unnecessary or excess materials that may result from various metabolic activities, cellular stress responses, or disruptions in homeostasis [26]. In this regard, this selective packaging ensures that specific proteins, lipids, and nucleic acids are released by endothelial cells in both physiological and pathological contexts, thereby facilitating intercellular signaling that can regulate endothelial function or contribute to endothelial dysfunction.

In a healthy endothelium, ECs release EVs that foster vascular and tissue homeostasis, ensuring a stable microenvironment crucial for overall health. EVs released by healthy ECs actively contribute to vascular repair, promote angiogenesis, and exert anti-inflammatory effects, which are critical in preserving endothelial integrity and supporting overall cardiovascular health. In contrast, during endothelial dysfunction, ECs release EVs that may develop a pathogenic profile when exposed to stress conditions, such as oxidative stress, chronic inflammation, or hyperglycemia. These vesicles may carry a pro-inflammatory and pro-thrombotic cargo, which, in turn, can perpetuate or exacerbate endothelial dysfunction through autocrine, paracrine, and endocrine signaling. This harmful cycle not only accelerates the decline in endothelial health but also contributes to the progression of CVD by creating a pro-inflammatory and pro-thrombotic environment [27].

## 4. Regulation of EV Communication in the Endothelium by Physiological Factors

### 4.1. Sex-Associated Differences in EV Profile and CVD Risk

It is widely recognized that men are at a greater risk of experiencing cardiovascular events compared with women of the same age, a phenomenon largely attributed to the protective effects of estrogen [28]. Additionally, testosterone can have harmful effects, further elevating the risk of CVD in men [29]. Beyond hormonal influences, unique male-specific pathophysiological mechanisms may further contribute to this increased susceptibility. The literature available on this topic is still evolving; nevertheless, a compelling study by Cai and colleagues sheds light on the issue [30]. They discovered that EVs isolated from the blood plasma of men with coronary artery disease contain DNA fragments of the SRY (sex-determining region, Y), absent in EVs from female patients and at higher levels than in the plasma of healthy men. Remarkably, EC exposure in culture to SRY-containing EVs led to increased expression of adherence factors such as ICAM-1 (intercellular adhesion molecule 1) [30]. Furthermore, in an in vivo model, administering SRY-containing EVs to *ApoE*^−/−^ mice accelerated the development of atherosclerosis [30]. This evidence underscores the existence of a potential male-specific pathway that could significantly contribute to cardiovascular risk by EVs.

In women, menopause represents a significant turning point in cardiovascular health. Postmenopausal women face a significantly higher risk of cardiovascular events compared to women of reproductive age, a shift that is often attributed to the loss of estrogens protective effects. Despite ongoing debate regarding the protective role of estrogen in cardiovascular health, strong evidence links menopause to an impairment in endothelial and vascular function—key contributors to CVD [31,32]. Chang et al., have uncovered the critical role of endothelial-derived EVs in this scenario, showing that the plasma of postmenopausal women is enriched with EC-derived EVs enriched with miRNAs linked to CVD [33]. Moreover, the Kronos Early Estrogen Prevention Study has shown that with declines in endogenous estrogen at menopause, the numbers of endothelium-, platelet-, monocyte- and granulocyte-derived microparticles increase [34,35]. In addition, Jayachandran et al. further highlighted that those women with low baseline cardiovascular risk showed increased circulating levels of MVs from activated endothelial and inflammatory cells. These MVs exhibited the expression of cell adhesion molecules and procoagulant factors, underscoring a dangerous pro-thrombotic state in postmenopausal women [34,35]. These altered EVs release, and the derived miRNAs may provide insight into the endothelial dysfunction prevalent in postmenopausal women, potentially clarifying their increased risk for cardiovascular complications

### 4.2. Impact of Senescence on EC-Derived EVs in CVD

Even in the absence of cardiovascular risk factors, individuals inevitably face functional cardiovascular decline as part of natural cellular senescence. Aging is a complex physiological phenomenon with significant pathological consequences, impacting various organs and systems, including the cardiovascular one. As we age, our cells undergo a series of detrimental changes, including oxidative damage resulting from the accumulation of reactive oxygen species (ROS), epigenetic modifications, and telomere shortening, which limits cell renewal and tissue repair [36,37]. These cellular changes contribute to a gradual decline in organ function and increase the risk of chronic diseases, with CVD being the most prevalent. Adopting a healthy lifestyle—such as engaging in regular physical activity, maintaining a balanced diet rich in antioxidants, and managing stress—can help delay these detrimental changes; nonetheless, they are ultimately unavoidable aspects of the cellular aging process. Recognizing and understanding these factors allows us to take proactive measures to enhance our longevity and quality of life as we journey through aging [38].

ECs, like any other cell in the body, can undergo senescence, losing their ability to divide and function properly. Senescent ECs play a critical role in the development of endothelial dysfunction and exhibit a unique secretory profile known as the senescence-associated secretory phenotype (SASP). This phenomenon significantly affects cardiovascular physiology, particularly concerning the development of atherosclerosis [39]. Besides the SASP, recent evidence has shown that senescent ECs also boost the production of EVs, particularly exosomes, which exhibit significant changes in the molecular cargo [40,41,42,43,44]. A study by Wong et al. reported the critical changes occurring in senescent ECs. They found that young, healthy human umbilical vein ECs (HUVECs) treated with exosomes derived from aged HUVECs exhibited significant reductions in growth and migratory abilities. This suppressed expression of specific markers indicates that EVs from senescent ECs play an active role in perpetuating endothelial dysfunction [43].

This mechanism becomes particularly concerning as localized endothelial dysfunction can become systemic issues through EV-mediated endocrine signaling pathways, potentially triggering a range of endothelial-related diseases. A critical example of this is advanced atherosclerosis, where the influence of EVs from senescent ECs becomes notably pronounced. In a compelling study by Alique et al. [45], MVs were extracted from the culture supernatant of ECs subjected to indoxyl sulfate, which induces geroconversion, a state of irreversible cellular senescence. When vascular smooth muscle cells (VSMCs) were exposed to these EVs, researchers documented a striking rise in calcium accumulation, alongside the significant upregulation of pro-calcification genes like *Runx2* and *BMP*, as well as inflammatory markers such as TNF-α, TWEAK, CCL2, CCL5, and IL-6 [45]. This evidence suggests that senescent EVs exacerbate vascular calcification and promote inflammation, thereby significantly contributing to the pathogenesis of atherosclerosis and other interlinked CVD.

## 5. EC Communication Through EVs Across Cardiovascular Risk Factors

### 5.1. Dyslipidemia

Elevated levels of lipids and lipoproteins in circulation (dyslipidemia) are a key driver of atherosclerotic plaque formation and progression [46]. Among the various lipoproteins, low-density lipoproteins (LDLs) are the most extensively studied due to their abundance and role as biomarkers of cardiovascular risk [47]. LDLs are major lipoproteins that transport cholesterol and other lipids throughout the body, supplying essential fats to cells. However, prolonged circulation of LDLs, particularly in the context of elevated levels that exceed the tissue incorporation capacity, leads to their oxidation. Oxidized LDL (ox-LDL) particles are recognized by macrophage scavenger receptors, facilitating their internalization into the subendothelial space as part of a homeostatic response. Monocytes, circulating leukocytes and an integral part of the phagocytic system, migrate to tissues upon activation signals, differentiating into macrophages with enhanced phagocytic activity [48]. Within the subendothelial space, macrophages that engulf ox-LDL undergo a phenotypic transformation into foam cells, initiating a pro-inflammatory cascade. This cascade encompasses the recruitment of fibroblasts and the commencement of vascular calcification, driving the growth of atherosclerotic plaques and the risk of cardiovascular events [49].

Moreover, the role of foam cells in inflammatory responses extends beyond the activation of immune cells, such as monocytes and macrophages; it also involves the modulation of EV secretion, which further impacts EC function and contributes to the progression of atherosclerosis [50]. Numerous studies have examined the responses of peripheral blood-derived human monocytic cells (THP-1) to exposure to ox-LDL and the implications for the development of atherosclerosis. Notably, EVs secreted from THP-1 exposed to ox-LDL are enriched with specific ncRNAs, such as LIPCAR and miR-106-3p, both of which are essential for vascular health. LIPCAR, a lncRNA, is known to drive endothelial dysfunction by inhibiting cell growth, inducing apoptosis, and increasing oxidative stress in ECs while promoting proliferation in VSMCs [51]. Furthermore, miR-106-3p is associated with enhanced cell proliferation and reduced apoptosis in VSMCs through the direct suppression of Caspase 9 [52]. These findings evidence the role of monocyte-derived EVs in the pathophysiological processes underlying atherosclerosis. Research conducted by Huang et al. provides substantial support for the hypothesis regarding the interactions between EVs released by ox-LDL-stimulated macrophages and EC functionality. They demonstrated that exposure of ECs to exosomes derived from ox-LDL-stimulated macrophages results in a notable reduction in EC proliferation and a significant impairment in their angiogenic potential [53]. These insights are valuable for elucidating the mechanisms through which ox-LDL-stimulated macrophages can influence EC function, either in a quiescent state within the circulation or during active infiltration into tissues.

On the other hand, EV-mediated intercellular communication in atherosclerosis may also occur from the endothelium to leukocytes. EC exposure to ox-LDL results in alterations to their secretory profiles, adopting a pro-inflammatory and pro-atherosclerotic phenotype. This shift suggests that EVs released by dysfunctional ECs in response to ox-LDL exposure could considerably influence the behavior of circulating inflammatory cells and contribute to the pathophysiology of atherosclerosis. Evidence from recent studies indicates that EVs secreted by ox-LDL-treated ECs can facilitate the formation of neutrophil extracellular traps (NETs). NET formation enhances tissue damage by promoting inflammation and thrombosis, which, in turn, worsens atherosclerotic conditions and increases the risk of plaque rupture and subsequent cardiovascular events [54]. The mechanisms whereby ox-LDL-treated EC-derived EVs induce NET formation are linked to the activity of specific ncRNAs, including MALAT-1 and miRNA-505, as demonstrated by independent studies [55].

### 5.2. Obesity

Obesity represents a prevalent risk factor for CVD in contemporary society. Individuals affected by obesity often adhere to high-fat diets, which leads to ectopic fat accumulation around the vessel wall and within critical vascular structures, such as the subendothelial space, contributing to the development of atherosclerotic plaques [56]. While the implications of obesity in CVD are frequently linked to increased lipid levels within the bloodstream, numerous studies have documented altered profiles of circulating EVs in obese patients, which may further aggravate endothelial dysfunction and cardiovascular risk.

Under typical physiological conditions, adipose tissue plays a critical role in sustaining vascular homeostasis through the secretion of bioactive molecules called adipokines. Additionally, adipose tissue contributes to maintaining vascular equilibrium by releasing proangiogenic EVs. These EVs are characterized by their low levels of miR-126, a microRNA recognized for its anti-angiogenic properties. MiR-126 operates by inhibiting the sprouty-related enabled/VASP homology 1 domain-containing protein (SPRED) complex, which is a known negative regulator of the MAPK/ERK signaling pathway—a critical pathway involved in cellular growth, proliferation, and differentiation [57]. However, in obese individuals, the proangiogenic properties of adipose tissue-derived EVs are significantly impaired. This decline derives from a reduction in the vesicular content of key factors such as the vascular endothelial growth factor (VEGF) and matrix metalloproteinases-2 (MMP-2), along with increased miR-126 expression [58]. Moreover, these obesity-altered EVs not only possess anti-angiogenic characteristics but also lead to detrimental changes in endothelial adhesion. A pivotal study conducted by Wadey et al. shows that adipocytes, when exposed to inflammatory and hypoxic conditions, secrete EVs that enhance the expression of vascular cell adhesion molecule-1 (VCAM-1) in vascular ECs. This effect is mediated through the activation of TNF receptor 1 (TNFR1) and the nuclear factor kappa B (NF-κB) pathway, while the classical MAPK/ERK pathway does not appear to be involved in this specific mechanism [59].

In addition, recent research has highlighted the critical role of the lncRNA-small nucleolar RNA host gene 9 (lncRNA-SNHG9) in the context of obesity-associated EVs. LncRNA-SNHG9 is essential for the lncRNA–mRNA interaction networks that are active during adipocyte differentiation, suggesting its potential involvement in the development of obesity. In this study, researchers described that exosomal SNHG9 derived from adipocytes can effectively mitigate inflammation and apoptosis in ECs by directly binding to and silencing the expression of TNF receptor-associated death domain (TRADD) mRNA. The knockdown of TRADD notably leads to a significant reduction in inflammation and apoptosis within ECs. Furthermore, the research highlights a concerning trend: exosomes isolated from the plasma of individuals suffering from obesity display markedly lower levels of SNHG9. This decrease is notably accentuated in patients exhibiting signs of endothelial dysfunction. This correlation potentially contributes to a cascade of biological events that trigger or worsen the endothelial dysfunction observed in this population [60].

### 5.3. Diabetes

Diabetes is a pathological condition characterized by the persistent dysregulation of blood glucose levels, either due to insufficient insulin production or diminished tissue sensitivity to the hormone. In both cases, blood glucose levels remain elevated (hyperglycemia), and an increased proportion of glycosylated hemoglobin (gHb) can be detected in circulation [61]. In the cardiovascular context, particularly in atherosclerosis, gHb is recognized by scavenger receptors on macrophages, which internalize it into the sub-endothelial space, thereby promoting the formation of atherosclerotic plaques. This phenomenon makes diabetes not only a pathology by itself but also an independent cardiovascular risk factor [62].

In addition to the well-documented impacts of glucose and gHb on the endothelium, circulating EVs may significantly contribute to the progression of endothelial dysfunction and the evolution of CVD in these patients. A study using a murine model of diabetes (db/db) found that diabetes impairs the reparative function of EC-derived EVs in the ischemic heart. This impairment occurs, at least in part, due to the transcriptional suppression of genes involved in angiogenesis, cell proliferation, and cell survival in the recipient cardiac ECs [63]. Furthermore, a recent study that combined metabolomics and proteomics delved into the mechanisms underlying EV-mediated endothelial dysfunction in patients with advanced diabetes, particularly those experiencing persistent complications. The findings indicated that EVs isolated from these patients promote the upregulation of the coagulation factor fibrinogen (FIBA) and ICAM-1, both of which are associated with endothelial dysfunction [64]. Nevertheless, despite the comprehensive proteomic and metabolomic analyses conducted to identify the pathways altered by EVs in ECs, these studies have not established how diabetes-induced metabolic changes affect the profile of these EVs. This gap highlights the need for further research to fully understand the complex interplay between diabetes and EV biogenesis and function in cardiovascular health.

To address this issue, studies have highlighted the impact of hyperglycemia on EV biogenesis and cargo content. For example, Wang et al. explored how circRNA-0077930, found in EVs from high-glucose-stimulated ECs, influences VSMCs. Their research demonstrated that circRNA-0077930 accelerates VSMC senescence by negatively regulating miR-622 expression, subsequently enhancing the pro-senescence gene *KRAS* [65]. In a related study, Zhang et al. established that monocytes exposed to elevated glucose levels secrete EVs that adversely affect endothelial function by delivering miR-142-5p. This particular miRNA inhibits insulin-like growth factor 1 (IGF1) signaling, resulting in diminished cell proliferation, migration, and angiogenesis [66].

In addition to the direct effects of hyperglycemia on ECs, other metabolic pathways altered in diabetes likely contribute significantly to modifying EV phenotypes. One key pathway involves arginase, an enzyme that catalyzes the conversion of arginine into ornithine and urea, playing a critical role in the urea cycle [67]. Elevated levels of arginase have been closely associated with compromised left ventricular function in patients following myocardial infarction [68,69]. Arginase also impacts microcirculation by depleting arginine, a crucial precursor of NO, thereby diminishing its bioavailability, which is essential for maintaining vascular function [70]. In the context of EVs, studies have shown that erythrocyte-derived EVs from patients with type-2 diabetes can carry arginase-1. These EVs have been found to induce endothelial dysfunction by reducing NO bioavailability in mouse aorta [71,72]. Moreover, research in diabetic mice has demonstrated that the transfer of arginase contained in EVs to ECs severely impairs their function, particularly as regards endothelial-mediated vascular relaxation and flow [73]. Consequently, the increase in arginase levels in the bloodstream and its direct adverse effects on ECs may significantly contribute to the deterioration of left ventricular function, further exacerbating cardiac issues in the diabetic population.

The relationship between glucose metabolism and changes in EC function extends beyond high glucose levels, as it persists even when diabetes and hyperglycemia are effectively managed pharmacologically. The lasting effects of high glucose levels on EC phenotypes, referred to as “hyperglycemic endothelial memory”, represent a significant aspect of vascular health that warrants attention. This memory is likely influenced by miRNAs [74,75] and could be communicated through EVs, highlighting the importance of understanding intercellular communication in the context of diabetes and its complications.

### 5.4. Metabolic Syndrome

Metabolic syndrome is a cluster of cardiovascular risk factors present in individuals, which may not necessarily manifest with overt symptoms but significantly increase the risk of cardiovascular events. These conditions include high blood pressure, elevated fasting glucose levels, elevated cholesterol, and excess body fat. Having at least three of these conditions qualifies as metabolic syndrome [76].

In individuals diagnosed with metabolic syndrome, there is a worrisome elevation of circulating microparticles, specifically EVs, which directly correlates with the increased cardiovascular risk associated with the various comorbid conditions of the syndrome. These EVs primarily originate from platelets, erythrocytes, and ECs, exerting a profound influence on endothelial function. When healthy ECs are exposed in vitro to EVs derived from individuals with metabolic syndrome, there is a detrimental reduction in nitric oxide (NO) production via the inhibition of endothelial nitric oxide synthase (eNOS) activity. Moreover, in vivo injection of metabolic syndrome EVs into mice impaired endothelium-dependent relaxation and decreased endothelial NO synthase expression [77].

The process of EV biogenesis and release is intricately influenced by the various metabolic conditions associated with metabolic syndrome. This complexity presents a significant challenge when attempting to identify specific cargo patterns in EVs that could be linked to metabolic syndrome. As a result, researchers are likely to find a diverse and heterogeneous collection of EV profiles, each contributing to endothelial dysfunction through a multitude of signaling pathways. Nonetheless, it is important to recognize that, despite the molecular diversity of these EV cargos, individuals suffering from metabolic syndrome consistently exhibit elevated levels of lipopolysaccharide (LPS)-enriched EVs in their circulating plasma [78]. This surge in LPS-enriched EVs has been implicated in the activation of the Toll-like receptor 4 (TLR4) pathway in EC [78], which plays a pivotal role in the inflammatory response. In addition, once LPS-enriched EVs activate this pathway, a marked increase in ROS generation was observed in both mitochondrial and cytosolic EC compartments. Activating the TLR4-mediated inflammatory and pro-oxidative signaling pathways has profound implications for endothelial health, as it may contribute to the development of endothelial dysfunction and significantly increase the risk of CVD in these patients.

## 6. Clinical Impact of EVs in CVD: Biomarkers for Diagnosis and Prognosis

Endothelial dysfunction is increasingly recognized as an important contributor to the onset and advancement of CVD, significantly influencing conditions such as atherosclerosis, hypertension, and heart failure. Among the CVDs, cardiac ischemic disease, particularly myocardial infarction, stands out as one of the leading causes of mortality and disability globally. It not only affects patients’ quality of life but also imposes staggering costs on healthcare systems [79,80]. Substantial advancements have been made in the management of myocardial infarction, including improved therapeutic protocols; nevertheless, the ability to predict outcomes—such as survival rates, the risk of developing heart failure, and the likelihood of future major adverse cardiac events related to maladaptive cardiac changes—continues to be a challenge. Moreover, current diagnostic strategies for evaluating coronary artery obstruction and assessing the risk of myocardial infarction predominantly rely on invasive procedures, such as cardiac catheterization (angiography). These procedures can be costly, uncomfortable for the patients, and occasionally yield inconclusive results, complicating clinical decision-making.

In this context, there is a pressing need for reliable biomarkers to detect subclinical atherosclerosis and to evaluate the severity of endothelial dysfunction that may lead to an increased risk of cardiovascular events. Furthermore, the inability to accurately predict long-term outcomes following myocardial infarction highlights the urgent need for new solutions. This is where EVs come into play. EVs have emerged as a highly innovative area of research in cardiovascular medicine and as promising biomarkers for tailored medicine. They can be collected relatively easily and are known for their stability in biofluids. Unlike traditional biomarkers, which often provide limited snapshots of pathological processes, EVs’ molecular composition and concentration change significantly in response to various cardiovascular conditions [81]. This dynamic characteristic allows for the capture of detailed insights into the cellular environment at different phases of disease progression, thereby making EVs valuable indicators for early diagnosis and prognosis.

The use of EVs as a diagnostic tool has garnered the most attention in the context of atherosclerosis. Numerous studies have established a strong correlation between specific miRNAs found in EVs and the presence of atherosclerotic plaques, suggesting that these biomolecules not only serve as indicators of plaque presence but also possess the ability to predict plaque vulnerability, which is critical for clinical decision-making. A study conducted in 2016 by Leistner et al. identified key miRNAs—miR-126-3p, miR-145-5p, miR-155-5p, and miR-29b-3p—as significant markers associated with advanced atherosclerosis. This research elucidated the differential expression levels of these miRNAs in the plasma of patients, demonstrating notable variations between those presenting with stable plaques and those with vulnerable plaques at risk of rupture [82].

The application of EVs as biomarkers can also significantly enhance the outcome prognosis following myocardial infarction. In this regard, characterizing circulating EVs in the aftermath of an acute ischemic event is fundamental, as these vesicles significantly mediate reparative processes in the heart [83]. Furthermore, the profile of these EVs may influence the development of adverse clinical events that can arise post-infarction [84].

Recent advancements in therapeutic management and rapid intervention have markedly reduced the rate of mortality associated with myocardial infarction. Consequently, this decrease has prompted a shift in focus toward managing subsequent post-infarction complications, with particular emphasis on heart failure.

Heart failure arises from maladaptive changes in the myocardium as the heart attempts to compensate for the loss of contractile tissue after ischemic injury. Moreover, it is increasingly recognized that EVs and their associated miRNA content can significantly influence heart failure pathophysiology. Increasing evidence has provided insights into the specific miRNAs present within these EVs, linking them to three principal processes associated with heart failure:

1. Cardiomyocyte hypertrophy: A variety of miRNAs have been implicated in cardiomyocyte hypertrophy, including, but not limited to, miR-1, miR-19, miR-21, miR-22, miR-26, miR-98, miR-101, miR-133, miR-145, miR-150, miR-199, miR-212, miR-221, miR-328, and miR-378. These miRNAs are instrumental in regulating cellular growth and adaptation in response to stress [84].
2. Myocardial fibrosis: The development of myocardial fibrosis, which contributes to stiffening of the heart muscle and impaired function, has been linked to specific miRNAs, such as miR-7, miR-16, miR-29, miR-30, miR-101, miR-125, and miR-133 [84].
3. Myocardial angiogenesis: The process of angiogenesis following a myocardial infarction, which is critical for restoring blood supply to the injured myocardium, involves various miRNAs, including miR-16, miR-31, miR-92, miR-126, miR-130, miR-143, and miR-214. These miRNAs have been recognized to promote new vessel formation and enhance tissue repair [85].

## 7. The Use of Healthy Endothelial EVs as a Potential Therapeutic Strategy in CVD

There is increasing interest in utilizing EVs as a therapeutic strategy for CVD, owing to their inherent capability to transport bioactive molecules between cells. Besides the ability to cross biological barriers, EVs possess several advantages over traditional drug delivery systems, including low immunogenicity and high biocompatibility [86]. EVs can boost treatment precision by directly delivering therapeutic agents—like drugs, proteins, and nucleic acids—to targeted cells. This targeted delivery maximizes the therapeutic impact while minimizing systemic side effects, thereby contributing to improved patient outcomes. Published and ongoing studies and clinical trials at different phases (I-III) are examining how stem cell-derived or bioengineered EVs can help treat various diseases. Although most studies are centered on cancer, some recent placebo-controlled clinical trials have demonstrated their potential to improve outcomes for patients with cardiovascular disease [87,88,89]. Additionally, EVs can be engineered to augment their targeting abilities to deliver tailored treatments, ensuring that therapeutic cargo is directed to specific tissues or cell types; these characteristics position EVs as a promising alternative for enhancing the precision and efficacy of medical treatments [86,90].

Among the various bioactive molecules found in EVs, miRNAs are particularly noteworthy due to their critical roles in cellular regulation. However, the independent administration of miRNAs presents inherent challenges and risks. Without the regulatory environment provided by living cells or EVs, the uncontrolled activity of miRNAs can increase the risk of undesirable side effects. An important illustration of this challenge can be found in the study conducted by Gabisonia and colleagues [91], which aimed to employ miRNA-199 therapy to promote myocardial regeneration in the porcine model of myocardial infarction. While the regeneration of the myocardium unfolded as anticipated, the actions of miRNA-199 were not effectively controlled, resulting in the improper innervation of the newly formed tissue, leading to malignant arrhythmias and underscoring the risks associated with unregulated miRNA therapies.

Another advantage of using EVs for miRNA-based interventions is the complexity associated with their delivery to target areas. Currently, the typical approach for miRNA delivery involves encapsulation within lipid nanoparticles; however, these nanoparticles tend to accumulate in the liver rather than reaching the intended target tissues. This unintended distribution significantly diminishes their therapeutic efficacy and raises the potential for adverse effects associated with off-target delivery, particularly in the liver [92]. In light of these challenges, current research efforts are being directed toward standardizing the production of EVs for clinical applications. This includes developing immortalized cell lines for large-scale EV production, establishing scalable methodologies for isolating and purifying these vesicles, and implementing rigorous quality assessments to ensure their safety and efficacy for patient use [93,94].

Focusing specifically on endothelium-derived EVs, compelling evidence supports their efficacy in modulating repair processes within the heart after a myocardial infarction. Numerous studies have demonstrated that the administration of EC-derived EVs to damaged heart tissue significantly enhances vascularization surrounding the borders of the infarcted area, which is crucial for restoring blood flow and promoting healing. Additionally, these interventions have been shown to improve overall cardiac function, potentially limiting the long-term maladaptive remodeling following myocardial infarction [95,96]. Endothelial colony-forming cells (ECFCs), a specialized subset of ECs, have emerged as a promising source for obtaining autologous EC-derived EVs [97]. Their potential advantages in myocardial infarction therapy are well documented, providing a strong foundation for future research aimed at harnessing EVs as a novel therapeutic approach to enhance cardiovascular health and improve patient outcomes.

While extracellular vesicle (EV)-based therapies demonstrate remarkable therapeutic potential, they face substantial hurdles that stem from challenges in purification and large-scale production. Although numerous EV purification methods have been developed, a universally accepted gold-standard protocol for isolating EVs from biofluids with high purity and yield has yet to be established. Common protocols involve sequential centrifugation, starting with a medium-speed spin to remove cell debris, followed by high-speed centrifugation to pellet MVs and resuspension in an appropriate physiological buffer. Exosome isolation; however, is far more challenging due to their small size, lower concentration, and the limited number of proteins and miRNAs they carry, often overshadowed by the abundant protein content in biofluids [98]. Advanced protocols often require the extensive ultracentrifugation of diluted samples paired with size-exclusion chromatography to achieve more effective exosome isolation. Combining these approaches can significantly reduce contamination from proteins that are eluted at different rates based on their size. However, it is important to note that these methods cannot ensure total purity, as other EVs and contaminants may also fall within the same size range [99].

In addition to purification, scaling up the production, storage, and distribution of EVs while maintaining their quality and functionality poses a considerable challenge in current biotherapeutic development. The standard methods for EV production typically rely on large-scale cell culture systems, which necessitate the use of robust, scalable bioreactors. These systems must not only support cell viability but also ensure consistent and efficient EV production while minimizing the risks of contamination. The specific source of the donner cells utilized significantly impacts EV composition and therapeutic efficacy, making it critical to maintain consistent cell lines and precisely controlled culture conditions for producing EVs with predictable and reproducible properties [100,101].

Despite these challenges, ongoing research and technological advancements in areas such as cell culture optimization, isolation techniques, and characterization methods offer promising avenues for overcoming these current limitations. By strategically addressing these challenges, we can unlock the full transformative potential of EVs as a transformative class of therapeutics.

## 8. Conclusions

The accumulating evidence in EV research supports the notion that these macro and nano extracellular particles are key players in the progression of endothelial dysfunction. The molecular cargo within EVs not only highlights key pathways in the progression of endothelial dysfunction but also presents key elements in the cross-talk between the endothelium and surrounding tissues in health and disease. In this regard, a comprehensive understanding of EV profiles associated with endothelial dysfunction and CVD, including atherosclerosis and heart failure, is essential to translate these insights into clinical practice and boost the diagnostic and therapeutic potential of EV biology in cardiovascular health.
